# Supplementary material for: Using Mobile Phone Sensor Technology for Mental Health Research: Integrated Analysis to Identify Hidden Challenges and Potential Solutions
Source: J Med Internet Res. 2018 Jul 30;20(7):e10131. doi: 10.2196/10131 (PMC6090171; doi:10.2196/10131)
Supplement: Multimedia Appendix 1 [file jmir_v20i7e10131_app1.pdf]

Entry survey

Q1: How old are you?

A1: 18-24, 25-34, 35-44, 45-54, 55-64, 65 and over

Q2: What is your gender?

A2: Male, female, other

Q3: How much time do you spend on your mobile phone on average in a day?

A3: Less than 30 minutes, from 30 minutes to 1 hour, from 1 to 2 hours, from 2 to 3 hours

Q4: How old is your phone?

A4: 0-1 year, 1-2 years, 2-3 years, more than 3 years.

App performance survey

Q1: In the last week, did you experience any problems when running the app?

A1: Yes, no

Q1a: What issues did you experience?

A1a: Optional open question

Q2: In the last week, did you change any settings on your phone?

A2: Yes, I turned off Bluetooth; Yes, I turned off GPS; Yes, I turned off Mobile Data; Yes, I turned on Airplane Mode: Yes, I turned on Do Not Disturb; No, I did no change any of these settings

Q3: In the last week, did you use Bluetooth on your phone?

A3: No, Bluetooth headset, Bluetooth earphones, handsfree for car, fitness trackers like Jawbone/Fitbit, other

Q4: In the last week, did the app impact the battery life of your phone?

A4: 1, not much; 2; 3; 4; 5; 6; 7, very much

Q5: In the last week, did the app impact the data usage of your phone?

A5: 1, not much; 2; 3; 4; 5; 6; 7, very much

Ethics questionnaire

Q1: How comfortable were you with having your GPS data collected?

A1: Very comfortable, comfortable, neither comfortable nor uncomfortable, uncomfortable, very uncomfortable

Q1a: Can you tell us more about how you felt about having your GPS data collected? Why were you comfortable or not comfortable with this aspect of the study?

A1a: Optional open question

Q2: How comfortable were you with having your Bluetooth connections collected?

A2: Very comfortable, comfortable, neither comfortable nor uncomfortable, uncomfortable, very uncomfortable

Q2a: Can you tell us more about how you felt about having your Bluetooth data collected? Why were you comfortable or not comfortable with this aspect of the study?

A2a: Optional open question

Q3: How comfortable were you with having the app installed on your phone and running in the background?

A3: Very comfortable, comfortable, neither comfortable nor uncomfortable, uncomfortable, very uncomfortable

Q3a: Can you tell us more about how you felt about having the app installed on your phone and running in the background? Why were you comfortable or not comfortable with this aspect of the study?

A3a: Optional open question

Q4: How comfortable were you with filling out self-report questionnaires?

A4: Very comfortable, comfortable, neither comfortable nor uncomfortable, uncomfortable, very uncomfortable

Q4a: Can you tell us more about how you felt about filling out self-report questionnaires? Why were you comfortable or not comfortable with this aspect of the study?

A4a: Optional open question

Q5: Would you continue to use this app if it was available to you after the completion of this study? Why or why not?

A5: Optional open question

Q6: How comfortable are you, in general terms, with social media data collection for health / medical research?

A6: Very comfortable, comfortable, neither comfortable nor uncomfortable, uncomfortable, very uncomfortable

Q7: How comfortable are you, in general terms, with social media data collection for advertising and market research?

A7: Very comfortable, comfortable, neither comfortable nor uncomfortable, uncomfortable, very uncomfortable

Q8: How comfortable are you, in general terms, with social media monitoring in order to deliver evidence-based targeted medical interventions?

A8: Very comfortable, comfortable, neither comfortable nor uncomfortable, uncomfortable, very uncomfortable

Q9: How comfortable are you, in general terms, with social media monitoring for targeted legal interventions

A9: Very comfortable, comfortable, neither comfortable nor uncomfortable, uncomfortable, very uncomfortable

Q10. If you said that you were uncomfortable with any of the above practices, what concerns do you have about social media data collection or social media monitoring?

A10: Optional open question
